# Assessment of total, ligand-induced peroxisome proliferator activated receptor γ ligand activity in serum

**DOI:** 10.1186/s12940-019-0486-2

**Published:** 2019-05-09

**Authors:** Lariah Edwards, James Watt, Thomas F. Webster, Jennifer J. Schlezinger

**Affiliations:** Department of Environmental Health, Boston University School of Public Health, 715 Albany Street, R-405, Boston, MA 02118 USA

**Keywords:** Metabolism disrupting compounds, PPARγ, Human serum, Mixtures

## Abstract

**Background:**

Humans are exposed to a complex mixture of environmental chemicals that impact bone and metabolic health, and traditional exposure assessments struggle to capture these exposure scenarios. Peroxisome proliferator activated receptor-gamma (PPARγ) is an essential regulator of metabolic and bone homeostasis, and its inappropriate activation by environmental chemicals can set the stage for adverse health effects. Here, we present the development of the Serum PPARγ Activity Assay (SPAA), a simple and cost-effective method to measure total ligand activity in small volumes of serum.

**Methods:**

First, we determined essential components of the bioassay. Cos-7 cells were transfected with combinations of expression vectors for human PPARγ and RXRα*,* the obligate DNA-binding partner of PPARγ, along with PPRE (DR1)-driven luciferase and control eGFP reporter constructs. Transfected cells were treated with rosiglitazone, a synthetic PPARγ ligand and/or LG100268, a synthetic RXR ligand, to characterize the dose response and determine the simplest and most efficacious format. Following optimization of the bioassay, we assessed the cumulative activation of PPARγ by ligands in serum from mice treated with a PPARγ ligand and commercial human serum samples.

**Results:**

Cos-7 cells endogenously express sufficient RXR to support efficacious activation of transfected PPARγ. Co-transfection of an RXR expression vector with the PPARγ expression vector did not increase PPRE transcriptional activity induced by rosiglitazone. Treatment with an RXR ligand marginally increased PPRE transcriptional activity in the presence of transfected PPARγ, and co-treatment with an RXR ligand reduced rosiglitazone-induced PPRE transcriptional activity. Therefore, the final bioassay protocol consists of transfecting Cos-7 cells with a PPARγ expression vector along with the reporter vectors, applying rosiglitazone standards and/or 10 μL of serum, and measuring luminescence and fluorescence after a 24 h incubation. Sera from mice dosed with rosiglitazone induced PPRE transcriptional activity in the SPAA in a dose-dependent and PPARγ-dependent manner. Additionally, human serum from commercial sources induced a range of PPRE transcriptional activities in a PPARγ-dependent manner, demonstrating the ability of the bioassay to detect potentially low levels of ligands.

**Conclusions:**

The SPAA can reliably measure total PPRE transcriptional activity in small volumes of serum. This system provides a sensitive, straightforward assay that can be reproduced in any cell culture laboratory.

## Introduction

Historically, the rising incidence in obesity has been attributed to genetic background, changes in diet and the modern lifestyle, yet these factors fail to fully explain the rapid onset of the epidemic [1]. The commercial chemical registry instituted by the Toxic Substances Control Act currently consists of 84,000 chemicals [2]. At a minimum, 25,000 of these chemicals are actively used in commercial products today [3]. Exposures to these chemicals arise in the outdoor environment from industrial releases and the indoor environment from use of consumer goods. Widespread exposure to low doses of a number of these metabolism disrupting chemicals (MDCs), a class of endocrine disrupting chemicals (EDCs), has been linked to obesity due to their abilities to inappropriately activate fat-forming pathways and enhance weight gain through white fat accumulation [4].

The search for MDCs that act on adipose tissue has focused on identifying ligands for the nuclear receptor peroxisome proliferator activated receptor gamma (PPARγ). PPARγ regulates white, brown, and brite (brown-in-white) adipogenesis, mature adipocyte maintenance, function and survival, and insulin sensitivity [5–7], as well as the balance of adipogenesis and osteogenesis [8, 9]. PPARγ is a ligand-activated transcription factor that binds to the PPAR response element (PPRE, 5′-CAAAACAGGTCANAGGTCA-3′) heterodimerized with the retinoid x receptors (RXR) to regulate gene transcription [10]. Activation of PPARγ by exogenous ligands induces adipogenesis [11] and increases subcutaneous and abdominal adiposity [12, 13]. Additionally, PPARγ activation suppresses bone differentiation [14], resulting in bone marrow adiposity and reduced bone quality in mouse models [15, 16] and in humans [17, 18].

PPARγ ligands include structurally diverse endogenous and exogenous (natural and synthetic) ligands. Prostaglandins are known fatty acid-derived endogenous PPARγ ligands derived from the lipid membrane [19]. Exogenous ligands include naturally-occurring, therapeutic, and environmental chemicals. Natural products, such as foods and medicinal plants, containing PPARγ activating constituents have been investigated for their potential therapeutic potential [20]. Drugs of the thiazolidinedione class (e.g. rosiglitazone, Avandia®) are highly potent PPARγ agonists that upregulate PPARγ’s insulin sensitizing functions [11, 21]. While thiazolidinediones activate PPARγ with potencies in the mid-nanomolar range, natural PPARγ ligands and endogenous compounds have micromolar potencies [11, 20, 22].

Environmental PPARγ ligands are a growing class of MDCs, including phthalates, organotins, brominated flame retardants, organophosphate flame retardants and polycyclic aromatic musks, which induce adipogenesis [23–29]. Environmental PPARγ ligands have potencies ranging from nanomolar (i.e., organotins) to micromolar (i.e., phthalates) [24, 25, 27]. In addition to inducing adipocyte differentiation in vitro, at least phthalates [30], organotins [31, 32], and organophosphate esters [33] also increase adiposity in vivo. Additionally, growing evidence suggests that reduced bone quality is an adverse health effect of environmental PPARγ ligands [34–36].

Detection of environmental PPARγ ligands in human biological samples [37–41], confirms widespread exposure to these chemicals. People are exposed to a complex mixture of both environmental and therapeutic PPARγ ligands, and components of these mixtures — full agonists, partial agonists and competitive antagonists — act cumulatively to activate PPARγ in a predictable manner [42]. Nuclear receptor-dependent bioassays can provide inexpensive and rapid assessments of the biological activities of ligand mixtures. Originally developed to measure the aryl hydrocarbon receptor activity of polyhalogenated aromatic hydrocarbons [43], chemical-activated gene luciferase (CALUX) bioassays have been adapted and validated for use as tools to assess exposure to environmental ligands, including aryl hydrocarbon receptor, estrogen receptor and PPARγ ligands [44–50]. Here, we have developed a bioassay to quantify total PPARγ ligand activity in serum samples, using a rodent model and human serum samples from commercial sources. As PPARγ forms an obligate, permissive heterodimer with RXR, and ligands for either receptor can lead to signaling [51], we paid careful attention to the role of both receptors.

## Materials and methods

### Materials

Rosiglitazone (cat. #71740) was from Cayman Chemical (Ann Arbor, MI). DMSO (in vitro, cat. #AB03091) was from American Bioanalytical (Natick, MA). LG100268 (cat. #SML0279), T0070907 (cat. #T8703), DMSO (in vivo, cat. #D1435), and carboxymethylcellulose (cat. #C9481) were from Sigma-Aldrich (St. Louis, MO). HX 531 (cat. #3912) was from Tocris Bioscience (Bristol, UK). All other reagents were from Thermo Fisher Scientific (Suwanee, GA), unless specified.

### Animal studies

Animal studies were approved by the Institutional Animal Care and Use Committee at Boston University and performed in an American Association for the Accreditation of Laboratory Animal Care accredited facility (Animal Welfare Assurance Number: A3316–01). Care was taken to minimize distress. Nine-week-old, female, C57BL/6 J mice (RRID:IMSR_JAX:000664, Jackson Laboratories, Bar Harbor, ME) were treated by oral gavage (10 μL/g) with vehicle (Vh, 1% carboxymethylcellulose, 0.1% DMSO), or rosiglitazone (0.1, 1, 10, 100 mg/kg). One hour after gavage, mice were euthanized, and blood was collected via cardiac puncture. This time was chosen as it is the point of maximum serum concentrations following oral administration in humans [52, 53]. Serum was separated and frozen at − 80 °C.

### Human sera samples

Human AB serum samples were purchased from commercial sources (Atlanta Biologicals, Flowery Branch, GA; Corning Cellgro, Tewksbury, MA; Gemini Bio-products, West Sacramento, CA; MP Biomedicals, Santa Ana, CA; Sigma-Aldrich, St. Louis, MO). These sera were collected in the United States and were from men.

### Reporter assays

Cos-7 cells (RRID: CVCL_0224**,** CRL-1651, ATCC, Manassas, VA) were maintained in Dulbecco’s Modified Eagle’s Medium (DMEM) (Corning Cellgro, Tewskbury, MA) with 5% fetal bovine serum (FBS), amphotericin B/penicillin/streptomycin, and L-glutamine at 37 °C and 5% CO_2_ atmosphere. Cells were plated in 96-well plates in antibiotic free media and transiently transfected with expression vectors, individually or in combination, containing human *PPARG1* (provided by V.K. Chatterjee, U. Cambridge) [54], or human *RXRA* (plasmid 8882; Addgene, Cambridge, MA) [7] with PPRE × 3-TK-luc (plasmid 1015; Addgene) [55] and CMV-eGFP (mammalian expression vector for expression of green fluorescent protein) (from our laboratory) reporter constructs. The sequence of the PPRE is 5′-GTCGACAGGGGACCAGGACAAAGGTCACGTTCGGGAGTCGAC-3′). Transfected cultures were incubated overnight, and the media was replaced. Naïve wells were transfected with the reporter constructs alone and an empty pcDNA 3.1 plasmid (in proportion to the expression vectors) and left untreated. For chemical experiments, standard curve wells containing 100 μL of medium were treated with Vh (DMSO, 0.5%), rosiglitazone (10^− 10 ^–2 x 10^− 6^ M), or LG100268 (10^− 10 ^–2 x 10^− 6^ M). For serum experiments, standard curve wells containing 100 μL of medium received 10 μL charcoal-dextran stripped FBS and then were treated with Vh or standard curve chemicals. Experimental wells containing 100 μL of medium were treated with 10 μL of mouse serum in duplicate. Human serum was initially titrated at volumes ranging from 0.5–50 μL to determine proper volume. In final human serum experiments, wells were treated with human serum in duplicate or quadruplicate (10 μL). Referent (ref) wells containing 100 μL of media received 10 μL of stripped fetal bovine serum. In antagonist experiments, wells were treated with serum and then co-treated with Vh (DMSO:Ethanol, 50:50, 0.5%), T0070907 (1 μM) or HX 531 (2 μM). After a 24 h incubation, cells were lysed using Steady-Luc Firefly HTS Reagent (Biotium, Freemont, CA). Luminescence (lum) and fluorescence (eGFP or flo) were measured using a Synergy2 multi-function plate reader (Biotek, Inc. Winooski, VT). eGFP values were analyzed to ensure that no treatment caused a significant decrease in fluorescence, as this is an indicator of toxicity. Data were normalized using a method to minimize intra- and inter-experimental variation [56], as follows:

$$ Percent\ Positive\ Control\ Activity=\frac{\mathrm{Exp}\ \left(\frac{\mathrm{lum}}{\mathrm{flo}}\right)-\mathrm{Naive}\ \left(\frac{\mathrm{lum}}{\mathrm{flo}}\right)}{\mathrm{Positive}\ \mathrm{Control}\ \left(\frac{\mathrm{lum}}{\mathrm{flo}}\right)-\mathrm{Naive}\ \left(\frac{\mathrm{lum}}{\mathrm{flo}}\right)} $$

When subtraction of the Naïve (lum/flo) resulted in a negative number, the negative number was replaced with “0.” Concentrations in the plateau of the dose response curves were used to generate the positive controls. Rosiglitazone-treated cultures (10^− 7^–2 x 10^− 6^ M) served as the positive control for PPARγ-transfected cultures. LG100268-treated cultures (10^−6 ^ M) served as the positive control for RXR-transfected cultures. “PPRE transcriptional activity” is reported as the “% Maximum Activity,” relative to the positive control. To interpolate rosiglitazone concentrations in the mouse serum, the standard curve data were fit with the “log(agonist) vs. response (3 parameters)” function in conjunction with the “Interpolate unknowns from standard curve” function in Prism 6 (GraphPad Software, La Jolla, CA).

### Immunoblotting

Cos-7 cells were grown and maintained as described above. Whole cell lysates were prepared from Cos-7 cells and Cos-7 cells transfected with human *PPARG1*, human *RXRA,* mouse *Pparg1* (plasmid 8886; Addgene), mouse *Pparg2* (plasmid 8865; Addgene) [7], mouse *Rxra,* mouse *Rxrb,* or mouse *Rxrg* (provided by R. M. Evans, Salk Institute for Biological Studies) [57]. Cos-7 cells were washed twice with PBS, lysed in Cell Lysis Buffer (Cell Signaling Technology, Danvers, MA) and sonicated. The lysates were cleared by centrifugation, and the supernatants were used for protein expression analyses. Protein concentrations were determined by the Bradford method [58]. Total proteins (15–80 μg) were resolved on 10% gels, transferred to a 0.2 μM nitrocellulose membrane, and incubated with primary antibody (monoclonal rabbit anti-PPARγ (cat. #2443, Cell Signaling Technology) or monoclonal rabbit anti-RXRα (cat. #3085, Cell Signaling Technology)). Immunoreactive bands were detected using HRP-conjugated antibodies (cat. #7074, Cell Signaling Technology) followed by enhanced chemiluminescence. To assess protein loading, blots were re-probed with a β-actin-specific antibody (cat. #A5441, Sigma-Aldrich).

### Statistical analyses

Statistical analyses were performed with Prism 6 (GraphPad Software). For in vivo exposures, data are reported from individual mice (*n* = 4–12). In reporter assays, serum samples were run in duplicate. Standard curves were fit with “log(agonist) vs. response (3 parameters)” function. Data are reported as means ± standard errors. Student’s t-tests and ANOVA (one and two factor) combined with Tukey-Kramer, Sidak’s and Dunnett’s multiple comparisons tests were used to determine statistical significance. All analyses were performed using α = 0.05.

## Results

**Fig. 1:**
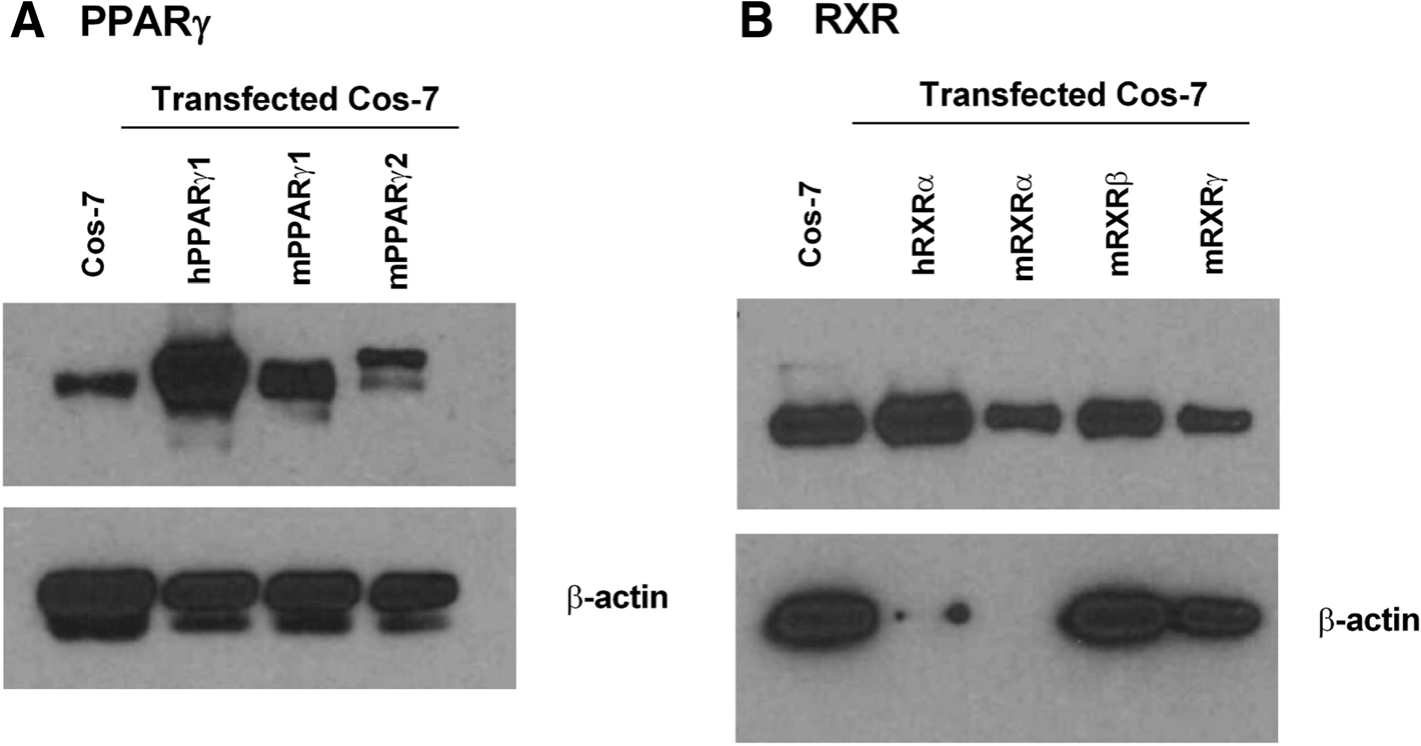
Cos-7 cells endogenously express PPARγ1 (A) and RXRs (B), proteins necessary for PPARγ to activate transcription. Whole cell lysates were prepared from Cos-7 cells and Cos-7 cells transfected with (**a**) mouse *Pparg1*, mouse *Pparg2*, human *PPARG1* expression vectors*,* or (**b**) human *RXRA,* mouse *Rxra,* mouse *Rxrb,* mouse *Rxrg* expression vectors*.* Lysates were analyzed for PPARγ, RXRα, and βactin expression by immunoblotting

First, we began by determining the essential elements needed to produce a robust reporter system. PPARγ binds to and activates transcription at PPREs as an obligate heterodimer with RXRs. To develop a biomarker of total serum PPARγ ligand activity, we began by examining the expression of PPARγ and RXRs in Cos-7 cells, the cell model for the reporter assay. Cos-7 cells endogenously express PPARγ and RXRs, the receptors necessary to support ligand-induced PPARγ activity (column 1 (left most), Fig. 1a-b). Expression of PPARγ could be augmented by transfection with an expression vector for human *PPARG1* (column 2, Fig. 1a). The additional 30 amino acids for the PPARγ2 isoform (column 3 vs 4, Fig. 1a) retards the migration of the protein and comparison demonstrates that our transfection protocol specifically increases the expression of PPARγ1. While the specific compliment of RXRs could not be identified because the isoforms co-migrate, it is evident that Cos-7 cells express at least one isoform that co-migrates with human and mouse RXRs (Fig. 1b).

**Fig. 2:**
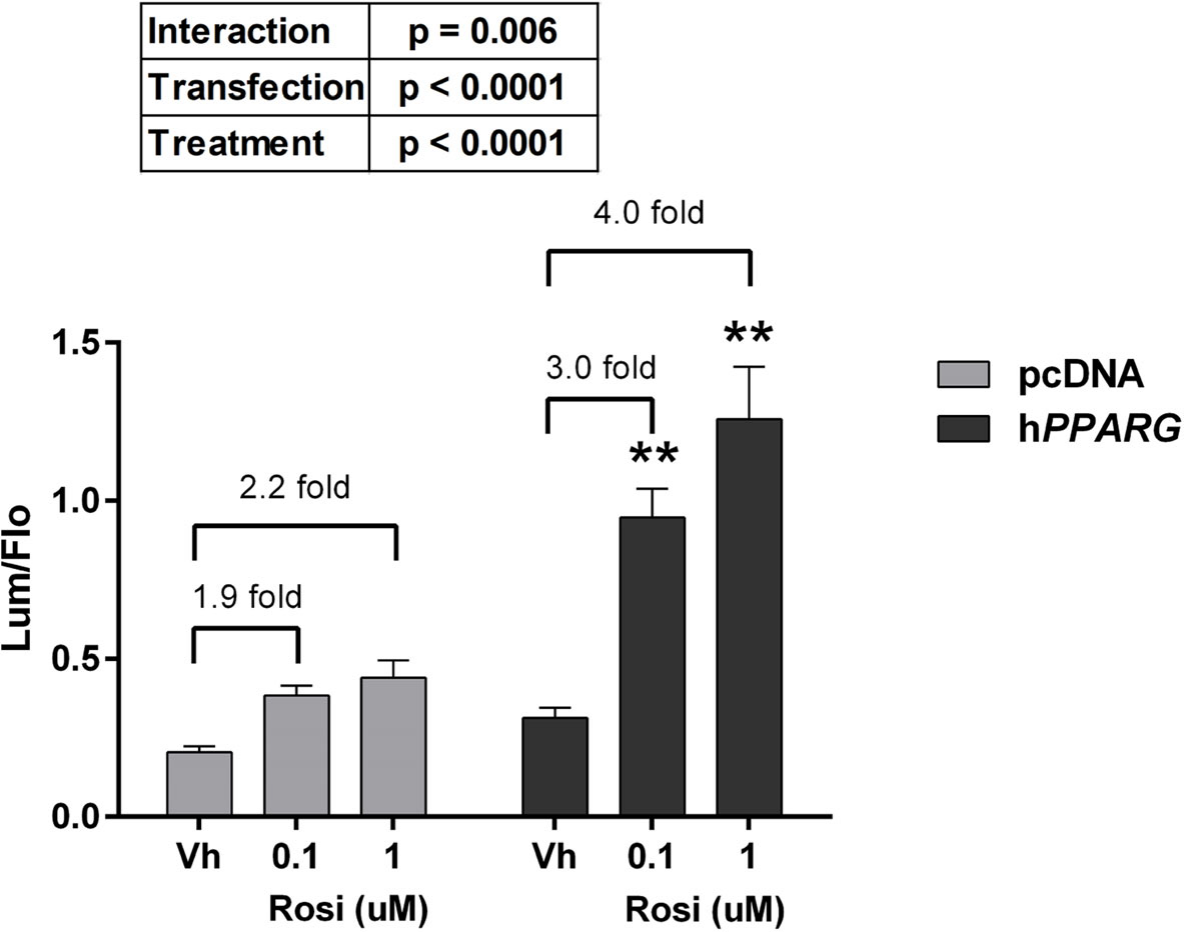
Overexpression of PPARγ significantly increases transcriptional response to rosiglitazone. Cos-7 cells were transfected with reporter plasmids (PPRE-luciferase reporter, CMV-GFP reporter) and empty pcDNA 3.1 or a mouse *Pparg2* expression vector. Cells then were treated with Vh (0.5% DMSO) or rosiglitazone (as indicated). Luminescence and fluorescence were measured after 24 h. Data are reported as mean ± standard error (*N* = 7 independent transfections). Statistical analyses indicated in the box are from a 2-Factor ANOVA. ** Significantly different from Vh (*p* < 0.01, ANOVA, Dunnett’s)

**Fig. 3:**
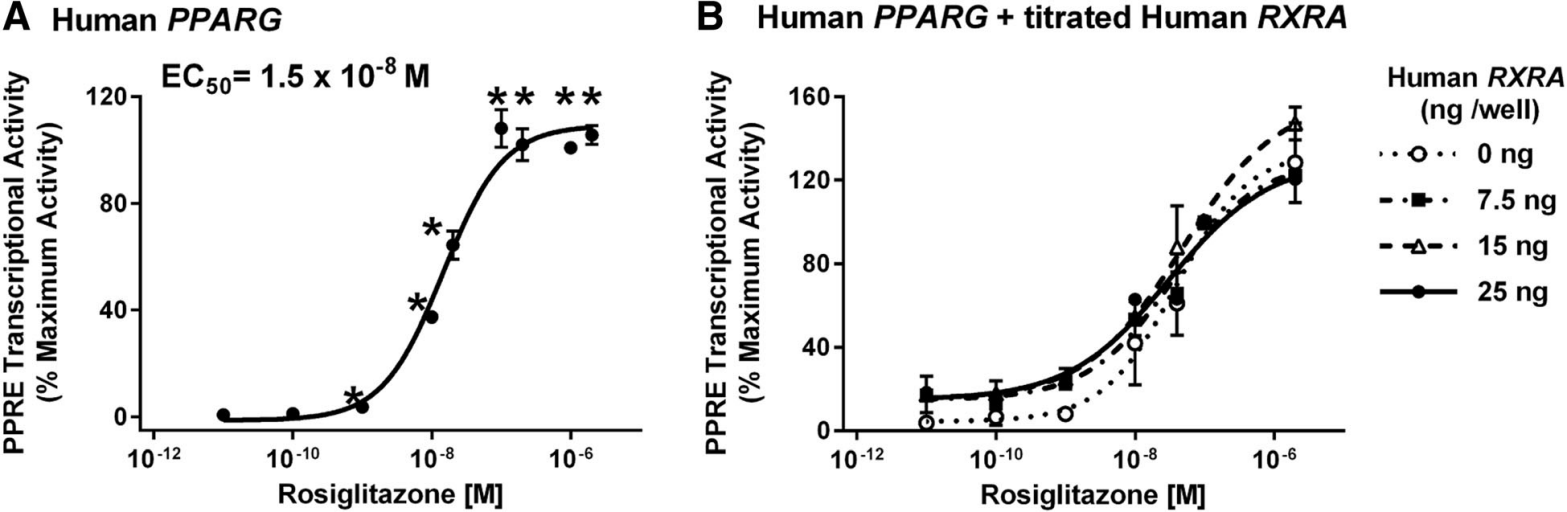
Overexpression of human PPARγ1 alone is sufficient to support robust PPARγ transcriptional activity. Cos-7 cells were transfected with reporter plasmids (PPRE-luciferase reporter, CMV-GFP reporter) and a human *PPARG1* expression vector (**a**) or human *PPARG1* and human *RXRA* expression vectors (**b**). Cells then were treated with Vh (0.5% DMSO, shown as 10^− 11^ M) or rosiglitazone (10^− 10^- 2 × 10^− 6^ M). Luminescence and fluorescence were measured after 24 h. Data were calculated as described in the Methods. Dose response data are reported as mean ± standard error (*N* = 3–4 independent transfections). Data were fit with a 3-parameter sigmoid equation. * Significantly different from Vh (*p* < 0.05, ANOVA, Dunnett’s)

Next, we determined the efficacy of activation of a PPRE-driven reporter in human *PPARG1*-transfected Cos-7 cells. First, we tested if increasing the expression of PPARγ would improve the signal to noise ratio in the assay. Rosiglitazone, a PPARγ-specific ligand [11], only significantly induced transcriptional activity in human *PPARG1*-transfected cells (Fig. 2). Second, we tested the rosiglitazone dose response for activation of PPARγ1. Prior to normalization by the positive control, the lum/flo values ranged from a minimum of ≈ 1 to a maximum of ≈ 50. As expected, rosiglitazone is a potent and efficacious ligand in this assay (Range: 10^−10^–2 x 10^− 6^ M, EC_50_: 1.5 × 10^− 8^ M)(Fig. 3a). We determined if the activity level could be increased further by co-transfecting with an *RXRA* expression plasmid. Co-transfection of increasing amounts of human *RXRA* expression plasmid with human *PPARG1* did not increase induction of PPARγ-dependent transcriptional activity by rosiglitazone (Fig. 3b).

**Fig. 4:**
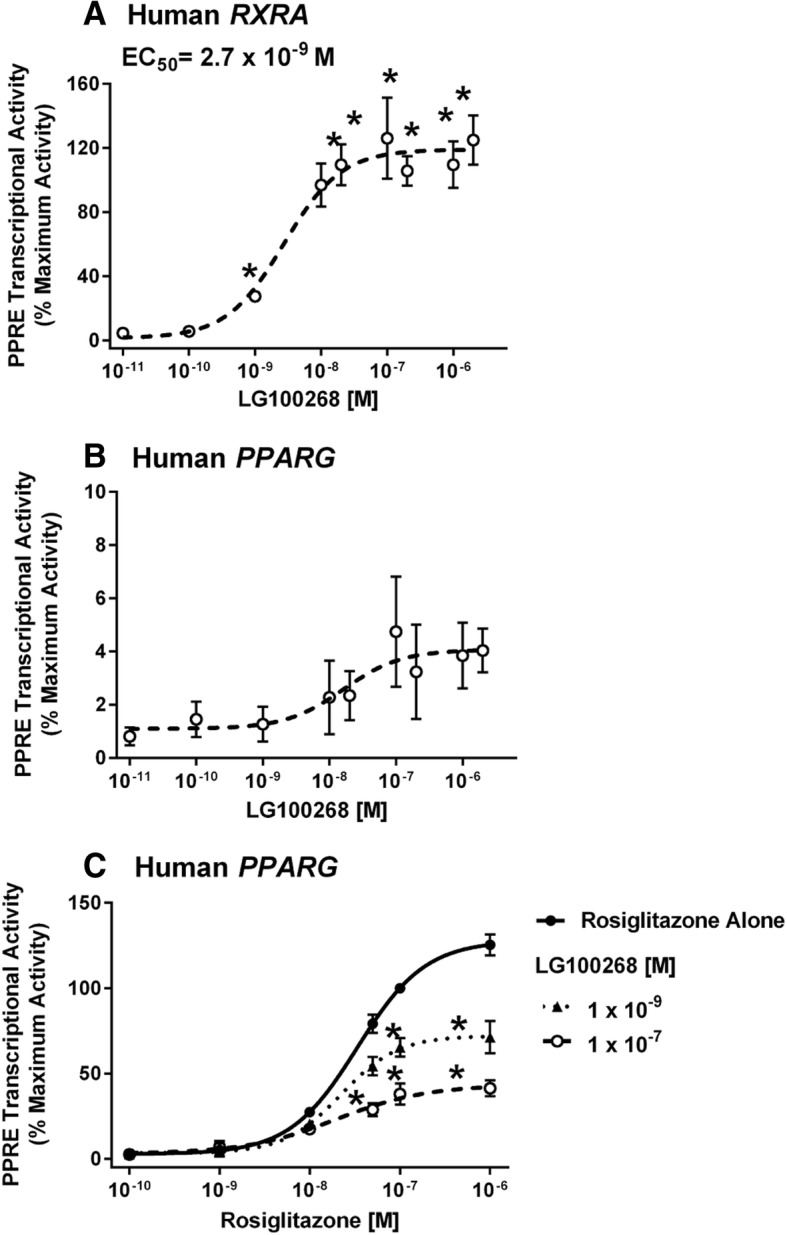
RXR ligands do not significantly activate PPARγ, but reduce rosiglitazone-induced, PPRE-dependent transcriptional activity. Cos-7 cells were transfected with reporter plasmids (PPRE-luciferase reporter, CMV-GFP reporter) and a human *RXRA* expression vector (**a**) or a human *PPARG1* expression vector (**b-c**). Cells were treated with Vh (0.5% DMSO, reported as 10^− 11^ M), rosiglitazone (10^− 10^-2 × 10^− 6^ M), and/or LG100268 (10^− 10^- 2 × 10^− 6^ M). Luminescence and fluorescence were measured after 24 h. Data were calculated as described in the Methods. Data are reported as mean ± standard error (*N* = 4 independent transfections) * Significantly different from Vh (*p* < 0.05, ANOVA, Dunnett’s).

The transcriptional activity of PPARγ can be activated by binding of a ligand to PPARγ itself or by binding of a ligand to its partner RXR in some cell types [51, 59]. Furthermore, RXR:RXR homodimers are known to activate transcription at PPREs [60]. Therefore, we examined the potential contribution of RXR activation to the PPRE transcriptional activity we were measuring. First, LG100268, an RXR-specific ligand [61], potently induced PPRE-dependent reporter activity when Cos-7 cells were transfected with human *RXRA* alone (EC_50_ = 2.7 × 10^− 9^ M)(Fig. 4a). To compare the efficacy of transcription at the PPRE through RXR vs PPARγ, we compared the lum/flo values. For PPARγ, the maximal lum/flo is ≈ 50, and for RXR, the maximal lum/flo is ≈ 5. Therefore, PPARγ is approximately 10-fold more efficacious at activating transcription at this PPRE. LG100268 did not significantly induce PPRE-dependent reporter activity when Cos-7 cells were transfected with human *PPARG1* alone (Fig. 4b).

According to Schulman et al. (1998), co-exposures to PPARγ and RXR ligands induced synergistic activation of transcriptional activity at PPREs when both *PPARG* and *RXR* expression vectors were transfected into 3 T3 L1 cells. We therefore assessed the potential interaction of PPARγ and RXR ligands in our assay. Contrary to expectation, treatment of human *PPARG1* transfected Cos-7 with increasing concentrations of LG100268 decreased rosiglitazone-induced transcriptional activity (Fig. 4c).

Overall, rosiglitazone induced robust PPARγ-dependent activity with the transfection of human *PPARG1* alone. LG100268, an RXR specific ligand, did not by itself induce activity, as is optimal for a PPARγ transcriptional assay. However, LG100268 reduced the rosiglitazone-induced activity, suggesting that the assay may produce an underestimate of the PPARγ activity if RXR ligands are present in significant quantities in a mixture of ligands.

**Fig. 5:**
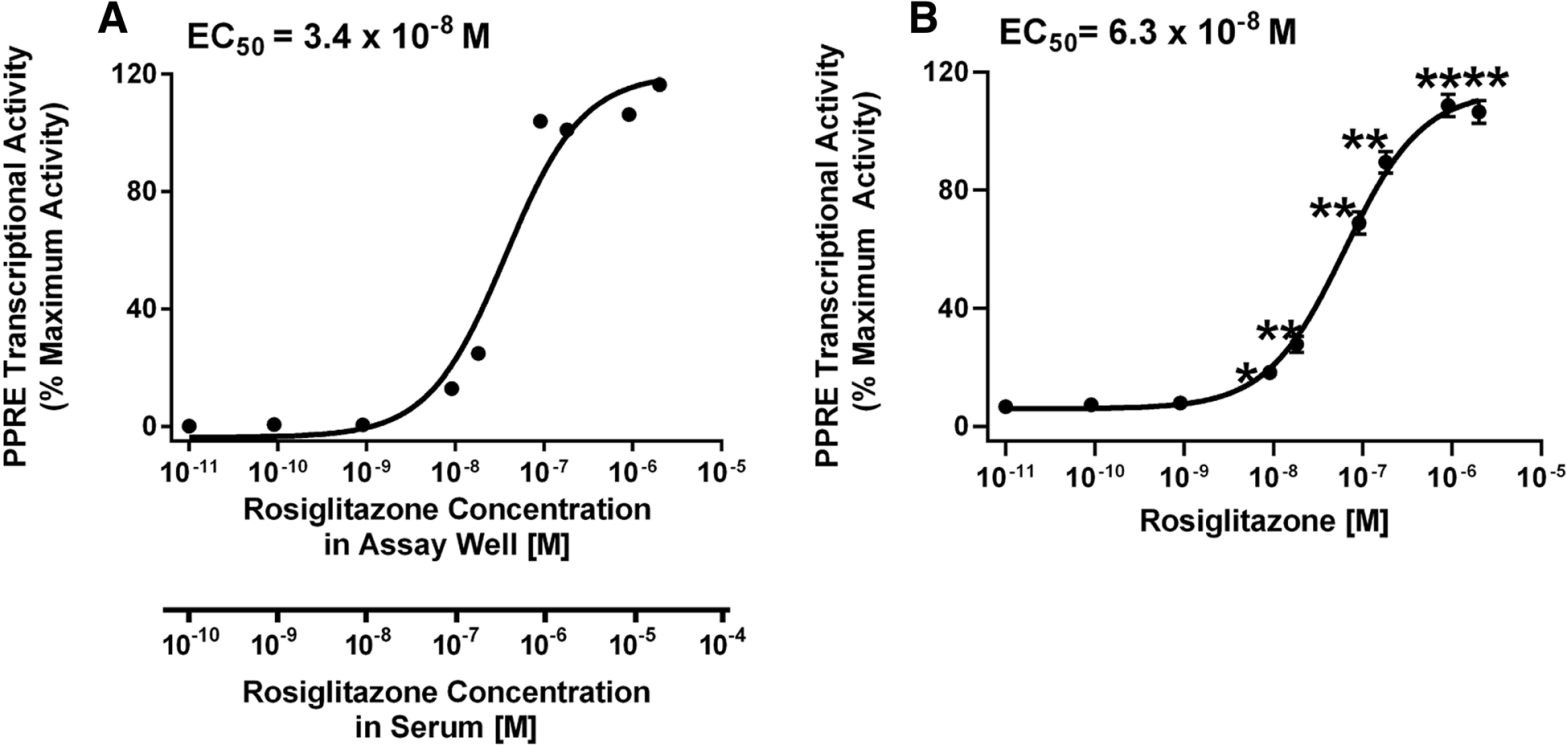
Standard curve for rosiglitazone in the Serum PPARγ Activity Assay with added serum. Cos-7 cells were transfected with reporter plasmids (PPRE-luciferase reporter, CMV-GFP reporter) and a human *PPARG1* expression vector. Charcoal stripped serum (10 μl) was added to each well and then the wells were treated with Vh (0.5% DMSO, shown as 10^− 11^ M) or rosiglitazone (10^− 10^ - 2 × 10^− 6^ M), in duplicate. Luminescence and fluorescence were measured after 24 h. Data were calculated as described in the Methods. Data were fit with a 3-parameter sigmoid equation. (**a**) Standard curve used to calculate mouse serum rosiglitazone concentrations. The upper X axis indicates the concentrations of rosiglitazone in the total volume in the assay well (10^− 10^-2 × 10^− 6^ M). The lower X axis indicates the concentrations of rosiglitazone in the volume of excess serum added to the well (10^− 9^- 2 × 10^− 5^ M). (**b**) Compilation of standard curves performed between 2016 and 2019. Data are reported as mean ± standard error (*N* = 29 independent transfections). Significantly different from Vh (* *p* < 0.05, **p < 0.01, ANOVA, Dunnett’s)

Second, we tested the efficacy of the assay in detecting a PPARγ ligand in serum. Increasing serum concentrations have been shown previously to reduce the apparent potency of ligands by reducing bioavailability [62]. We therefore generated a standard curve for rosiglitazone in the assay (transfected with h*PPARG1*), adding 10 μL charcoal-stripped FBS (Fig. 5). As expected, rosiglitazone has a slightly higher EC_50_ of 6.3 × 10^− 8^ M compared to conditions without excess serum: EC_50_ = 1.5 × 10^− 8^ M (Fig. 3a). Since our goal is to measure PPARγ agonist activity in serum, we therefore used the standard curve in Fig. 5 for the analyses. We define the limit of detection (LOD) here as the lowest concentration yielding PPRE transcriptional activity that is significantly different from Vh: 9 × 10^− 9^ M (Fig. 5b). The true LOD may be lower. As we will test the activity of a small amount of added serum, it is important to adjust concentrations for dilution. For 10 μL of serum added to 100 μL already in the well, the final concentration will be 1/11 (9%) of that in the serum. The LOD for rosiglitazone in added serum thus is approximately 10^− 7^ M or 100 nM (see lower X axis, Fig. 5a).

**Fig. 6:**
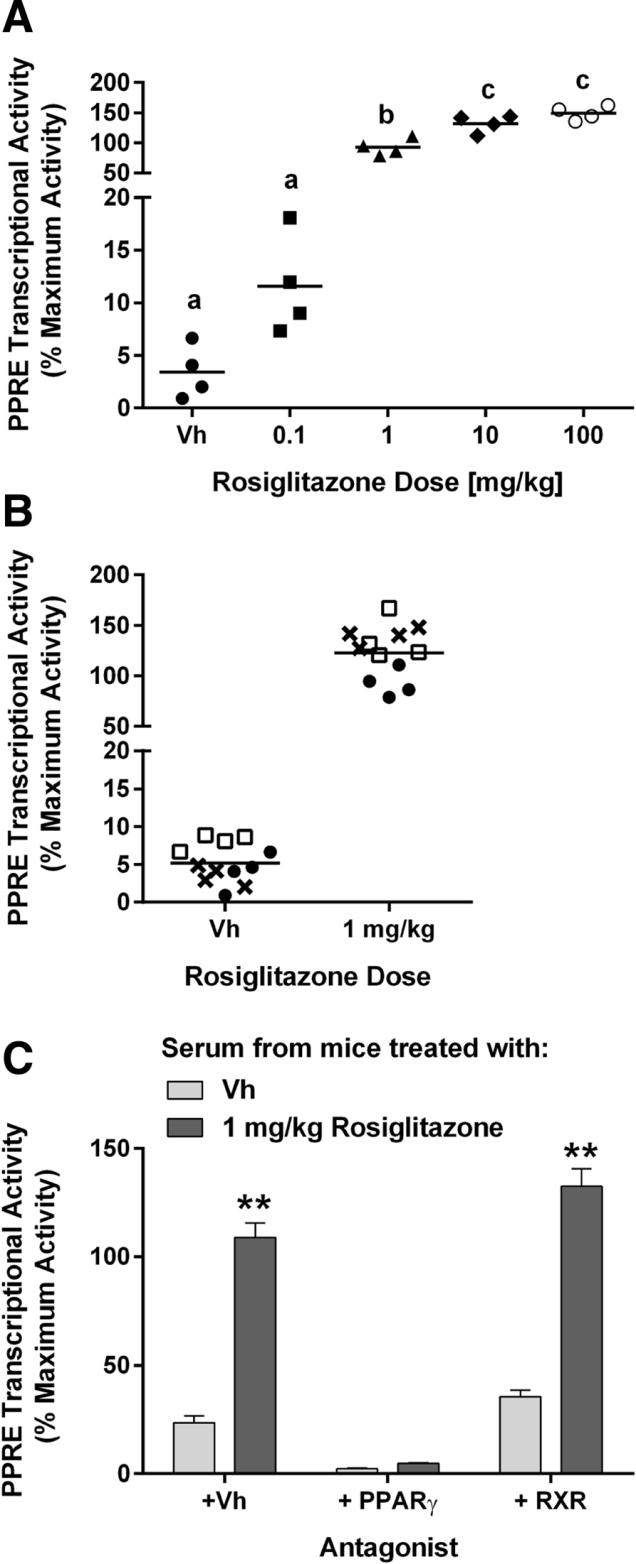
Serum PPARγ Activity can be detected in whole serum from rosiglitazone-treated mice in a dose-dependent manner. Sera were generated from nine-week-old, female, C57BL/6 J mice were treated by oral gavage with Vh (1% carboxymethylcellulose, 0.1% DMSO) or rosiglitazone (0.1, 1, 10, 100 mg/kg) and euthanized after 1 h. Sera were analyzed in Cos-7 cells transfected with reporter plasmids (PPRE-luciferase reporter, CMV-GFP reporter) and human *PPARG1*. Experimental wells were treated with 10 μL mouse serum, in duplicate. Luminescence and fluorescence were measured after 24 h. Data were calculated as described in the Methods and were calculated relative to the standard curve shown in Fig. 5. (**a**) Dose response of serum PPRE transcriptional activity. All mice were treated on the same day. (**b**) Reproducibility assessment. Mice were treated on three different days, with independently prepared dose solutions. Mice from each experiment are indicated by different symbols. For A and B, individual data are plotted with the mean indicated by a line. Different letters indicate group means that differed significantly, while groups with the same letter did not differ significantly (*p* < 0.05, ANOVA, Tukey). (**c**) To test for receptor specificity, wells treated with serum from mice that were exposed to Vh or to 1 mg/kg rosiglitazone were co-treated with Vh (0.5%, 50:50, DMSO:Ethanol), PPARγ antagonist (T0070907, 1 μM) or RXR antagonist (HX 531, 2 μM). For C, data are presented as means ± standard error from all mice in the treatment group. Significantly different from Vh (**p < 0.01, 2-Factor ANOVA, Sidak’s)

Serum likely contains endogenous PPARγ and RXR ligands that could potentially swamp or mask the activity of exogenous compounds. We therefore generated serum samples from C57BL/6 mice one hour after exposure via oral gavage to Vh or four doses of rosiglitazone. The serum was applied directly to Cos-7 cells transiently transfected with human *PPARG1*. Serum from Vh-treated mice had a significantly greater PPRE transcriptional activity than stripped FBS (5.2 ± 0.7 vs 0.1 ± 0.1% Maximum Activity, respectively; *p* < 0.006, Student’s t-test), thus there is likely endogenous or food-derived agonists present in mouse serum. Serum from rosiglitazone-treated mice activated PPARγ-dependent transcription in a dose-dependent manner. When analyzed using a Student’s t test, the lowest dose of rosiglitazone (0.1 mg/kg) also induced a significant increase in PPRE transcriptional activity relative to the background activity measured in Vh-treated mice (*p* < 0.02). Figure 6a shows the PPARγ activity associated with various doses; Fig. 5 provides the rosiglitazone concentrations associated with various levels of PPARγ activity. Taking into account volume dilution from adding serum to the assay, we estimate that the 0.1 mg/kg and 1 mg/kg doses resulted in mouse serum concentration of 55 nM (20 ng/ml) and 1.6 μM (580 ng/ml), respectively. Analysis of serum from mice treated with Vh or rosiglitazone on three separate days showed that the measured PPRE transcriptional activity was highly reproducible (Fig. 6b). The specificity of the assay for PPARγ-mediated PPRE transcriptional activity is shown by the complete abrogation of the serum-induced activity by T0070907, a PPARγ antagonist, but not HX 531, an RXR antagonist (Fig. 6c).

Overall, the results demonstrated that PPARγ agonist activity can be detected in whole serum, and that the activity is dose-dependent. Therefore, in the Serum PPARγ Activity Assay (SPAA), we proceeded with transfecting Cos-7 cells with human *PPARG1*, the PPRE reporter, and the control reporter (CMV-eGFP).

**Fig. 7:**
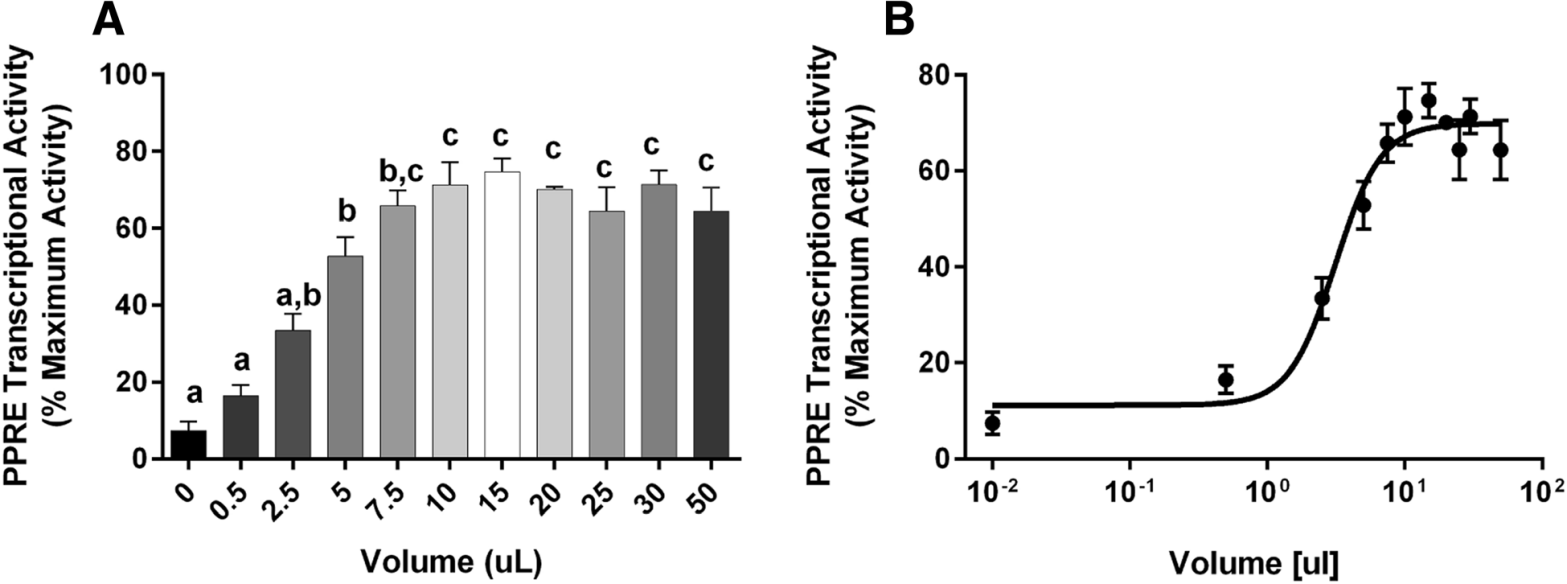
Determination of optimal serum volume in the SPAA. Cos-7 cells were transfected, and control wells were treated as described in Fig. 5. Experimental wells were treated with 0.5–50 μL of serum. Luminescence and fluorescence were measured after 24 h. Data were calculated as described in the Methods. (**a**) PPRE transcriptional activity measured in different volumes of serum (Gemini). Different letters indicate group means that differed significantly, while groups with the same letter did not differ significantly (*p* < 0.05, ANOVA, Tukey). (**b**) Dose response fit of PPRE transcriptional activity measured in different volumes of serum. Dose response data were fit with a 3-parameter sigmoid equation. Data are reported as mean ± standard error (*N* = 3 independent transfections)

Third, in order to determine the sensitivity of our bioassay to detect potentially low levels of PPARγ ligands in normal human serum, we examined the activation of PPARγ by commercial human serum samples. We began by titrating increasing amounts of human serum in the SPAA to determine the lowest volume that would provide a maximal signal. Increasing the volume of serum in the assay increased PPRE transcriptional activity (Fig. 7a), which was well fit by a sigmoid curve (Fig. 7b). We choose to use 10 μL of human serum because it was the lowest volume that induced a maximal PPARγ transcriptional activity across the human serum samples.

**Fig. 8:**
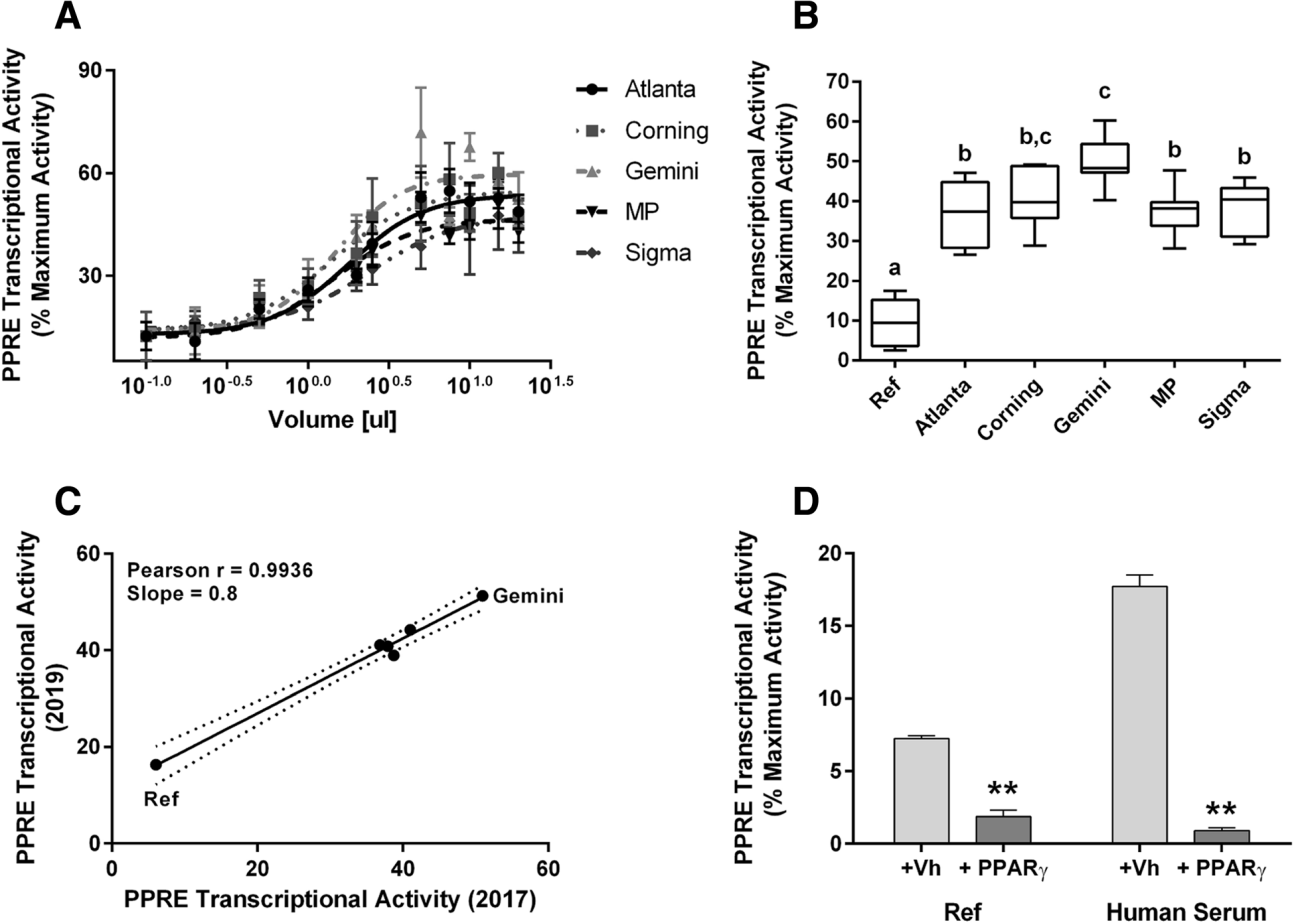
Commercial human serum samples from different sources have distinct PPRE transcriptional activity. Cos-7 cells were transfected with human *PPARG1* and treated with Vh or rosiglitazone standards as described in Fig. 5. Experimental wells were treated with 0.2–20 μL human serum. Luminescence and fluorescence were measured after 24 h. Data were calculated as described in the Methods. (**a**) PPRE transcriptional activity in different volumes of commercial human serum samples. Volume response data were fit with a 3-parameter sigmoid equation. (**b**) Comparison of PPRE transcriptional activity induced by 10 μL of commercial human serum samples. “Ref” refers wells that received 10 μL of stripped fetal bovine serum. Data are reported as mean ± standard error (*N* = 7 independent transfections; 4 runs in 2017 and 3 runs in 2019). Different letters indicate group means that differed significantly, while groups with the same letter did not differ significantly (*p* < 0.05, ANOVA, Tukey). (**c**) Correlation analysis of assay results from 2017 and 2019. (**d**) To test for receptor specificity, serum-treated wells were co-treated with Vh (0.5%, 50:50,  DMSO:Ethanol) or PPARγ antagonist (T0070907, 1 μM). Data are presented as means ± standard error from all human serum samples (*N* = 6) or 4 replicates of Ref serum. Significantly different from Vh (** *p* < 0.01, 2-Factor ANOVA, Sidak’s)

Comparison of the serum PPARγ agonist activity in five human serum samples purchased from different commercial sources showed that there is a range of detectable activity in normal human serum, which is dependent upon the volume of serum applied (Fig. 8a). The PPRE transcriptional activity induced by each sample at 10 μL was significantly different from the activity induced in the reference wells to which charcoal stripped FBS was applied (Fig. 8b). There were differences in the absolute values of PPRE transcriptional activity determined on different days (using distinct plasmid preps and cell passages) (Fig. 8c); however, the data were highly correlated even when samples were run years apart. The PPRE activity in the commercial human serum sample was reduced to the level of the reference samples (charcoal-stripped FBS) by a PPARγ antagonist, demonstrating the specificity of the assay (Fig. 8d). These data demonstrate the bioassay’s ability to detect a range of activities from unknown exogenous and endogenous ligands in small volumes of serum.

## Discussion

The growing concern with environmental chemical-mediated disruption of metabolism and obesity warrants improved methods to quantify exposure to environmental MDCs [4]. The traditional methods of assessing exposure to a single chemical fail to capture real world scenarios in which populations are exposed to multiple chemicals at a time, and these mixtures of chemicals may cause inappropriate activation of biological pathways. PPARγ plays a critical role in regulating lipid homeostasis, insulin sensitivity and bone homeostasis through the induction of target genes, thus making it a target for endocrine disruption by environmental MDCs [63].

**Fig. 9:**
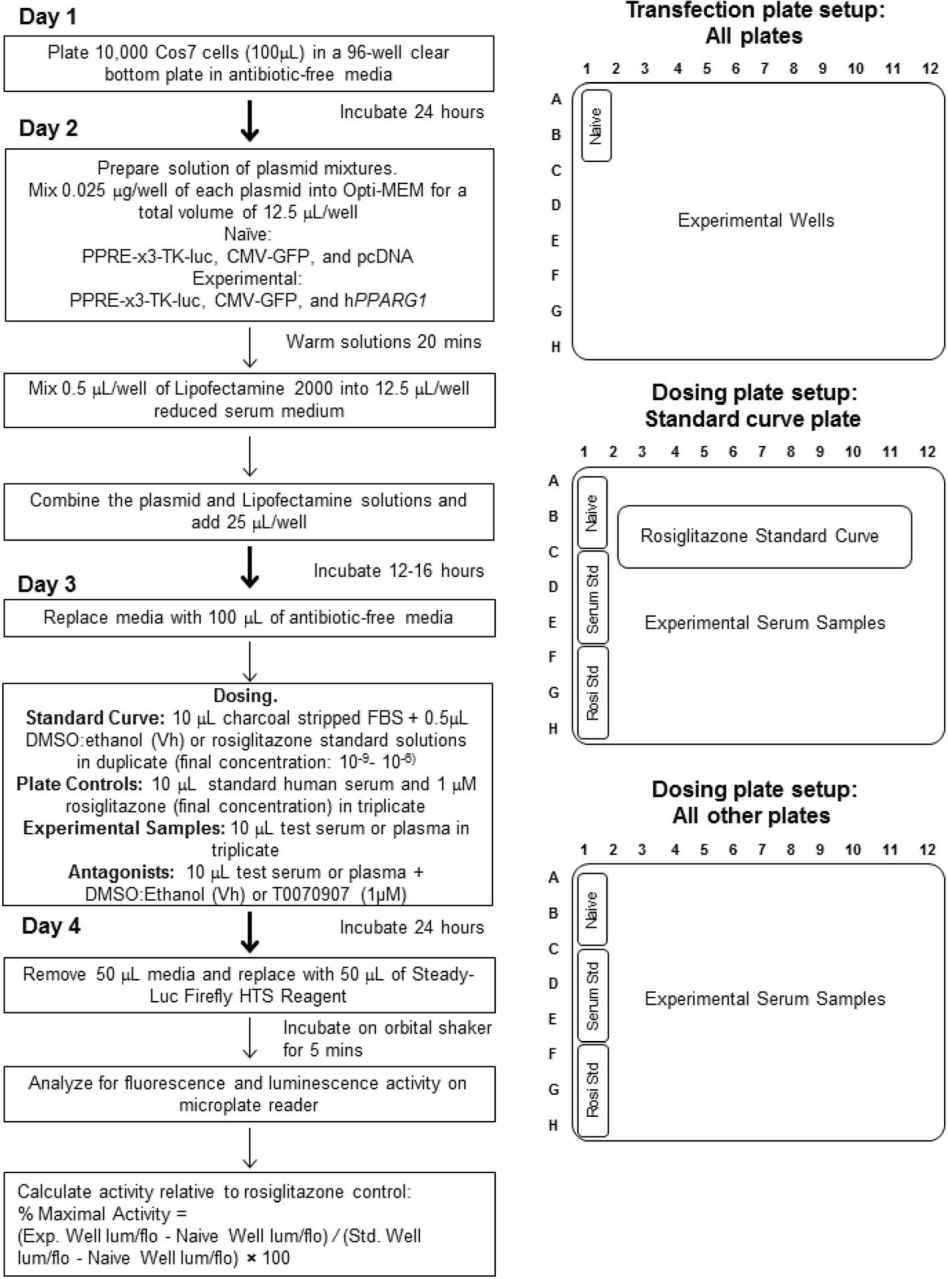
Flowchart and sample plate designs for performing the Serum PPARγ Activity Assay

The Serum PPARγ Activity Assay (SPAA) was developed to measure the total PPARγ activity of serum samples. SPAA uses Cos-7 cells transfected with PPARγ, a PPRE-driven reporter, and a constitutively active control reporter. We selected human PPARγ1 as the most appropriate ligand target because, compared to PPARγ2, PPARγ1 is more broadly expressed in tissues such as adipose, liver, kidney, muscle tissue, heart, and spleen [64, 65]. Table 1 provides information on all of the reagents needed to perform the assay. While only serum was tested in these analyses, the assay is expected to be compatible with plasma, as well. However, results from serum and plasma are not comparable, as they contain different concentrations of proteins that could affect the bioavailability of ligands. Figure 9 presents the assay protocol. To maximize reproducibility and for the most accurate comparison across samples, the same plasmid preparation, positive control solution and serum standard should be used to analyze samples. Additionally, Cos-7 cells at similar passage numbers should be used. Both positive controls and serum standards should be run on every plate. The serum standard could be used as an additional normalization factor for samples run with distinct plasmid preparations. Last, the receptor-specificity of the serum-induced activity can be determined by using antagonists to PPARγ and RXR as indicated in the protocol.

**Table 1:** Reagents to perform the Serum PPARγ Activity Assay

Reagents and Supplies	
Cos-7 cells (RRID:CVCL_0224, CRL-1651, ATCC) Maintenance media: Dulbecco’s Modified Eagle’s Medium (DMEM) (Corning Cellgro, Tewskbury, MA) with 5% fetal bovine serum (FBS), amphotericin B/penicillin/streptomycin, and L-glutamine 96-Well White-sided Clear Bottom Plates (cat. #: 07–200-566; Corning)	
Plasmids: human *PPARG1* [54] or mouse *Pparg1* (plasmid 8886; Addgene)^a^ PPRE-3x-Tk (plasmid 1015; Addgene) pcDNA (plasmid 20011; Addgene) CMV-eGFP (plasmid 11153; Addgene)^b^ Lipofectamine 2000 Transfection Reagent (cat. #: 11668019; Thermo Fisher Scientific) Opti-MEM Reduced Serum Media (cat. #: 31985062; Thermo Fisher Scientific)	
Charcoal/Dextran stripped FBS (cat. #: F6765, Sigma) Rosiglitazone (cat. #: 71740; Cayman Chemical) T0070907 (cat. #: T8703; Sigma) Vehicle solvent mix (50% Ethanol (cat. #: 04–355-453, Fisher Scientific): 50% Dimethyl sulfoxide (cat. #: AB03091; American Bioanalytical)) Commercial Human Serum Standard (cat. #: 100–512, Gemini Bio-Products)	
Steady-Luc Firefly HTS Assay (cat. #: 30028-L3; Biotium)	

^a^ We used human PPARγ1 provided by V.K. Chatterjee

^b^ We used CMV-eGFP made in house

Peak serum concentrations of rosiglitazone in humans occur one hour after oral administration [52, 53]. The maximum serum concentration of rosiglitazone in humans after receiving the lowest therapeutic dose (1 mg; approximately 0.1 mg/kg) is 76 ng/mL [66]; this is approximately equivalent to 200 nM in serum or 20 nM the assay well. At 1 h following gavage, serum from mice dosed with 0.1 mg/kg rosiglitazone induced PPRE transcriptional activity equivalent to 55 nM (20 ng/mL) rosiglitazone. The activity serum of mice treated with 0.1 mg/kg rosiglitazone was significantly greater than the activity measured in serum from Vh-treated mice, and the “endogenous” activity measured in the serum of Vh-treated mice was significantly greater than the activity in wells treated with stripped serum. SPAA thus is sensitive enough to detect PPARγ ligand activity in serum at levels below that induced by the lowest therapeutic dose in humans.

In vitro assays used alone or in combination with instrumental analyses, such as chromatography with mass spectrometry, increasingly have been used to detect and assess metabolic and endocrine disrupting chemicals. Previously developed CALUX bioassays have been used to measure PPARγ activity in food extracts, animal tissue, dust, and water samples [67–70]; these assays are available for purchase. However, RXR forms a permissive heterodimer with PPARγ and a ligand for either receptor can activate PPARγ-dependent transcriptional activity. Thus, RXR ligands, either endogenous (9-*cis* retinoic acid) or synthetic (rexinoids LG100268), can promote the transcription of PPARγ target genes [51, 59]. In the PPARγ CALUX validation study, it was speculated that some of the induced activity could be a result of the activation of endogenous RXR. Yet, the researchers did not directly consider the role of RXR [48, 67]. In our bioassay, an RXR ligand does not induce significant transcription of the PPRE-driven reporter when only *PPARG1* is transfected, a desirable feature of a PPARγ assay. It has been suggested that the combination of a PPARγ ligand and an RXR ligand can synergistically activate PPARγ-dependent transcription [51]. However, studies indicate that the permissive activity of the PPARγ - RXR heterodimer is largely dependent on cell type [71]. In our bioassay, increasing concentrations of a potent, synthetic RXR ligand reduced rosiglitazone-induced PPARγ transcriptional activity. However, endogenous, and natural RXR ligands (e.g. 9-cis-retinoic acid, phytanic acid, docosahexaenoic acid) have significantly lower potencies than LG100268 [72]. Thus, if RXR ligands are present in a serum sample, the activity of the PPARγ ligands may be underestimated; although the concentrations would have to be quite high for there to be a significant effect.

SPAA provides a measure of the combined activity of mixtures of PPARγ ligands in whole serum: a biological measure of exposure to mixtures containing full agonists, partial agonists and competitive antagonists. Generalized concentration addition (GCA) has been shown to accurately model the activity of mixtures of PPARγ ligands [42]. One possible use of SPAA is as a measure of exposure (biomarker of exposure) for epidemiology studies, particularly if combined with effect directed analysis to determine which mixture components are responsible for total activity [73, 74]. Alternatively, a combination of targeted instrumental analysis and GCA would yield an estimate of explained PPARγ activity that could be compared with total measured activity. For applications in epidemiological assessments, SPAA requires a very small volume of serum for testing (3 by 10 μL samples). Furthermore, this assay is cost effective and does not require any specialized equipment; most of the research materials can be found in a standard cell culture laboratory.

Importantly, SPAA is a first step in connecting PPARγ ligand exposure to adverse metabolic and bone health effects. Analysis of the distribution of serum-induced PPRE transcriptional activities could reveal the potential for exposure to environmental ligands. A normal distribution would suggest that the activity is largely derived from endogenous ligands, while a right-skewed distribution could indicate an environmental exposure or a therapeutic exposure. Identification of the chemical source of the activation would then be needed. White adipogenic, brite/brown adipogenic, insulin sensitizing and bone suppressing activities of PPARγ are regulated separately through differential post-translational modifications [21] and co-regulator recruitment [75], with ligands having distinct abilities to activate these PPARγ functions. Thus, the glitazone class of T2DM therapeutics (e.g., rosiglitazone and pioglitazone) activates PPARγ, stimulates brite adipocyte differentiation and increases insulin sensitivity [21], but improvement in metabolic health comes with significant side effects, including fat accumulation, hemodilution, cardiac hypertrophy and bone loss. Not surprisingly, these adverse effects also are mediated by PPARγ. We have shown that environmental ligands cause PPARγ to act in a biologically distinct manner. Because these ligands cannot trigger PPARγ to recruit the same coregulators they favor PPARγ’s ability to generate white adipocytes and to suppress bone formation [76, 77].

## Conclusions

A goal of our study was to develop an assay that can be reproduced in any cell culture laboratory. All the cells and reagents to perform this assay are publicly available, and the protocol is straightforward and uses common laboratory equipment. The results from this study lay the foundation for future work on the use of biomarkers of PPARγ ligand exposure that incorporate mixture effects. Our simple and cost-effective assay is both sensitive and specific to PPARγ ligands, including those present in whole serum samples. While this study did not characterize the components in serum that were driving activity, future work will investigate environmental chemical contributors to activity in a set of serum samples with well-documented exposure histories.

## Abbreviations

EDCs: Endocrine disrupting compounds
MDCs: Metabolism disrupting compounds
PPARγ: Peroxisome proliferator activated receptor-gamma
PPRE: PPAR response element
RXR: Retinoid x receptor
